# People living with HIV in Estonia: engagement in HIV care in 2013

**DOI:** 10.2807/1560-7917.ES.2016.21.43.30380

**Published:** 2016-10-27

**Authors:** Kaja-Triin Laisaar, Mait Raag, Irja Lutsar, Anneli Uusküla

**Affiliations:** 1 Institute of Family Medicine and Public Health, University of Tartu, Tartu, Estonia; 2 Institute of Biomedicine and Translational Medicine, University of Tartu, Tartu, Estonia

**Keywords:** human immunodeficiency virus, HIV, acquired immunodeficiency syndrome, AIDS, continuum of care, treatment cascade

## Abstract

Estonia had the highest rate of newly diagnosed human immunodeficiency virus (HIV) cases in the European Union (24.6/100,000) and an estimated adult HIV prevalence of 1.3% in 2013. HIV medical care, including antiretroviral therapy (ART), is free of charge for people living with HIV (PLHIV). To maximise the health benefits of HIV treatment, universal access should be achieved. Using data from surveillance and administrative databases and the treatment cascade model, we assessed the number of people infected with HIV, diagnosed with HIV, linked to HIV care, retained in HIV care, on ART, and with suppressed viral load (HIV-RNA: < 200 copies/mL). We identified that about one quarter of the 8,628 HIV-positive people estimated to live in Estonia in 2013 had not been diagnosed with HIV, and another quarter, although aware of their HIV-positive serostatus, had not accessed HIV medical care. Although altogether only 12–15% of all PLHIV in Estonia had achieved viral suppression, the main gap in HIV care in Estonia were the 58% of PLHIV who had accessed HIV medical care at least once after diagnosis but were not retained in care in 2013.

## Introduction

In 2013, Estonia had the highest rate of new human immunodeficiency virus (HIV) infections (24.6/100,000) and the third highest rate for acquired immunodeficiency syndrome (AIDS) diagnoses (1.8/100,000) in the European Union and European Economic Area (EU/EEA) [1]. The estimated HIV prevalence in the population aged 15–49 years was 1.3% [2]. People who inject drugs (PWID) have been disproportionately represented in the HIV-positive population since the beginning of the HIV epidemic in 2001 [1].

Estonia’s capacity to manage its response to HIV and AIDS has greatly increased over the past decade, particularly through initial funding from the Global Fund to Fight HIV/AIDS, Tuberculosis and Malaria in the mid 2000s [3]. In 2013, to confront the epidemic, the National HIV/AIDS Prevention Strategy for the period 2006 to 2015 was being implemented and medical care including antiretroviral therapy (ART) was free of charge for people living with HIV (PLHIV) throughout the study period, regardless of their medical insurance status [4]. In that period, HIV care was mainly provided by the government healthcare system through infectious disease clinics/departments in five major hospitals. Following the European HIV treatment guidelines, ART was recommended in 2013 for any HIV-positive person (without prior ART exposure) with a CD4^+^ T-cell count < 350 cells/mm^3^ [5]. For people with CD4^+^ T-cell counts above this level, when special conditions applied, ART was also carefully considered [5].

Potent combined ART has transformed HIV infection from an acute to a chronic disease and can reduce HIV transmission to HIV-uninfected partners [6]. Thus, availability of, and access to, ART are essential not only for individual, but can potentially also provide public health benefits [7]. Yet to maximise the health benefits of ART, health systems must ensure an effective cascade of high quality services provided to PLHIV [8].

The HIV/AIDS treatment cascade as a model to map PLHIV who actually receive the full benefit of the medical care they need for HIV, was first described by Gardner and colleagues [9]. Examining steps of the cascade allows to identify gaps in care for PLHIV and implement improvements. The cascade model has been applied in several countries to assess the performance of national healthcare programmes [10-12].

Our aim was to describe and quantify PLHIVs’ engagement with HIV/AIDS care in Estonia in 2013, applying the concept of the HIV care cascade.

## Methods

This was a cross-sectional review synthesising national-level HIV data, applying public health metrics for monitoring HIV care services, with focus on selected highest priority indicators.

### Data sources

We used the UNAIDS Spectrum estimate of the number of PLHIV in Estonia in 2013 [13]. Further, national databases were used to estimate the number of PLHIV at the next steps of the HIV treatment cascade in Estonia in 2013:

(i) The Estonian Health Board (EHB): An agency of the Estonian Ministry of Social Affairs (EMSA), responsible for passive surveillance of communicable diseases (including HIV), recording all newly diagnosed (confirmed by reference laboratory) HIV cases in Estonia; with nationwide coverage; data available online [14].

(ii) The Estonian Health Insurance Fund (EHIF): An institution operating within the administration area of EMSA as an independent legal body; the core purchaser of healthcare services for the compulsory health insurance system in Estonia, possessing healthcare utilisation data, covering all medical services and service costs (except ART medication costs) provided to PLHIV; with nationwide coverage. EHIF assigns each individual an identification code, enabling longitudinal tracking of care provided to individuals without personal identification (pseudo-identification). For study purposes special requests for data were submitted to EHIF.

(iii) the Estonian HIV Cohort Study (E-HIV): A database operated by the Estonian Society for Infectious Diseases which contains detailed and longitudinal demographic and clinical data of PLHIV in Estonia [15]. These include date of HIV confirmation, mode of HIV acquisition and course of HIV care (including dates of clinical appointments, ART provision, dates and results of CD4^+^ T-cell counts and viral load values) and concomitant diseases [15]. E-HIV was established in April 2009 and includes data from consenting PLHIV in HIV medical care; it also includes some retrospective data. For this study, data on 2,398 individuals with records from 1 September 2012 to 31 August 2013 were retrieved, whereas EHIF had records of 3,252 PLHIV for the same period.

(iv) In addition, data on vital events (AIDS related deaths) were obtained from the Estonian Causes of Death Registry (ECDR).

The specifications for computing the HIV cascade indicators retrieved from each of the databases are detailed in Table 1.

**Table 1:** Operational definitions for the six stages of the cascade of HIV care in Estonia, 2013

Stage	Operational definition, data with respective time period	Data source(s)
Living with HIV	The Spectrum estimate for 2013	UNAIDS
Diagnosed with HIV (alive in 2013)	Aggregated number of confirmed HIV-positive tests (individuals) minus the aggregated number of deaths (AIDS deaths, specified proportion of deaths related to illicit drug overdose) Time period: 1 January 1988^a^– 31 August 2013^b^	EHB; ECDR
Linked to HIV care (alive in 2013)	The number of individuals with at least one HIV-related healthcare visit, based on individual anonymised reimbursement claims of HIV-related healthcare services: visit dates, medical services provided to PLHIV (with dates), healthcare providers issuing the claims Time period: 3 February 2000^c^–31 August 2013^b^	EHIF
Retained in HIV care	The number of individuals with two or more HIV-related healthcare visits (at least three months apart) within the past 12 months Time period: 1 September 2012–31 August 2013^d^	EHIF
On ART	Step 1: The proportion of individuals on ART among those retained in care, based on individual anonymised data from E-HIV: HIV confirmation date, ART initiation date, dates and results of CD4^+^ T-cell and HIV RNA tests, dates of other provided medical services Step 2: The number of individuals on ART among those retained in care according to EHIF, when applying the proportion obtained in Step 1 to individuals retained in care according to EHIF Time period: 1 September 2012–31 August 2013^d^	E-HIV; EHIF
Virally suppressed	Step 1: The proportion of individuals on ART with the most recent (within the past 12 months) HIV RNA level < 200 copies/mL, based on individual anonymised data from E-HIV Step 2: The number of individuals virally suppressed according to EHIF, when applying the proportion obtained in Step 1 to individuals on ART according to EHIF Time period: 1 September 2012–31 August 2013^d^	E-HIV; EHIF

AIDS: acquired immunodeficiency syndrome; ART: antiretroviral therapy; ECDR: Estonian Causes of Death Registry; EHB: Estonian Health Board; EHIF: Estonian Health Insurance Fund; E-HIV: Estonian HIV Cohort Study; HIV: human immunodeficiency virus; PLHIV: people living with HIV; UNAIDS: Joint United Nations Programme on HIV/AIDS.

^a^ The first HIV case in Estonia was diagnosed in 1988.

^b^ End of our study period.

^c^ Earliest date appearing on an HIV-related medical service reimbursement claim (the date of opening the medical service account) in the EHIF electronic database since its inception.

^d^ To evaluate the situation in 2013, data from this 12-month period were used.

### Measures and definitions

For HIV treatment cascade indicators, we used the metrics developed by the United States Centers for Disease Control and Prevention (CDC) [16] and the Institute of Medicine (IOM) [17], adapted to Estonian data sources. Specifically we assessed: the number of persons (i) infected with HIV, (ii) diagnosed with HIV, (iii) linked to HIV care, (iv) retained in HIV care, (v) on ART, and (vi) with suppressed viral load (HIV RNA < 200 copies/mL). We also recorded whether PLHIV had started HIV medical care within three months of diagnosis [17]. We also looked at PLHIV CD4^+^ T-cell counts at different stages of HIV care to characterise their health state and the stage of the disease (HIV/AIDS).

We used only non-identifiable (anonymised or aggregated) data for this study, and the procedures met local data protection regulations.

#### PLHIV diagnosed with HIV

We obtained the number of PLHIV registered with the EHB from 1 January 1988 to 31 August 2013 [14]. In Estonia, patients have had to reveal their identity to get the HIV-positive preliminary/screening test result confirmed only since January 2009 [3]. From 2004 to 2008, 34% of new HIV cases were diagnosed anonymously at AIDS counselling centres, with 19% of individuals who tested positive admitting that they had had a positive test earlier [3]. Recording all these anonymous cases at the EHB probably caused some multiple registration of new HIV-positive cases [3]. In the current analysis, based on the five-year data from the AIDS counselling centres, we estimated that until 2009, altogether 6% (those anonymously testing positive and reporting having tested positive before) to 34% (those anonymously testing positive) of all the new HIV cases in Estonia may have been registered more than once.

To account for the deaths of PLHIV over time, we used national vital events statistics on the number of AIDS-related deaths. Due to role of PWID in the Estonian epidemic, we also considered the number of overdose-related deaths among HIV-positive PWID. To obtain an estimate for overdose-related deaths, we used the number of deaths with the International Statistical Classification of Diseases and Related Health Problems 10th revision (ICD-10) code X42: ‘accidental poisoning by and exposure to narcotics and psychodysleptics (hallucinogens), not elsewhere classified’ [18]; this accounted for the proportion of injected opiate deaths among the ICD-10 X42 deaths and the proportion of PWID ever having tested HIV-positive reported in local studies (data not shown).

Our 2013 estimate of the number of people diagnosed with HIV was therefore based on the number of people ever (between the first case in Estonia in 1988 and the end of our study on 31.08.2013) tested positive for HIV, and subtracting the number of AIDS-related deaths and a specified number of deaths from illicit drug overdose (in the same time period).

#### PLHIV linked to HIV care

To estimate linkage to and retention in HIV medical care, we used the following case-finding definition to obtain data from the EHIF database: all medical claims with HIV-related ICD-10 codes (B.20–B.24, F02.4, R75, Z21), including all healthcare service(s) provided to the patient(s) [18,19].

We estimated the number of HIV-positive people linked to HIV care by 2013, i.e. having ever accessed HIV/AIDS medical care in Estonia, from cumulative data from the healthcare services utilisation database of the EHIF from database inception till the end of our study (31 August 2013), only including data on PLHIV who were alive on 31 August 2013.

EHIF does not record data on HIV-related medical services provided to PLHIV in prison, as these services are financed through the Ministry of Justice. However, as only a few cases of HIV have been newly diagnosed in the detention system (personal communication: K. Kivimets, Estonian Ministry of Justice, 30 January 2014), we assumed that the majority of HIV-infected persons incarcerated on 31 August 2013, would have had at least one HIV-related contact with the medical care system before incarceration and would thus already be included in the EHIF database.

#### PLHIV retained in HIV care

Our estimate of retention in HIV care was based on data from the EHIF. Following the IOM definition for ’retained in HIV care’ [17], we considered PLHIV having had two or more consultations for HIV medical care (at least three months apart) within the past 12 month-period (1 September 2012–31 August 2013). Patients diagnosed less than three months before the end of the study period were excluded from this analysis.

#### PLHIV on antiretroviral therapy 

Data on PLHIV receiving ART were available from the E-HIV database. The proportion of PLHIV on ART was assessed among individuals considered ‘retained in care’ on 31 August 2013 and registered with E-HIV. This proportion was applied to the population ’retained in care’ according to EHIF to calculate the population-based ART coverage estimate.

#### PLHIV on ART with suppressed viral load

The proportion of individuals on ART in whom the virus was suppressed, was assessed from data from E-HIV. Individuals with their most recent HIV RNA level < 200 copies/mL during the study period (1 September 2012–31 August 2013) were considered to have achieved viral suppression. This proportion was applied to the population ’retained in care’ and on ART according to EHIF to calculate the population-based estimate.

In addition to estimating the six cascade steps, we also assessed the timing of initiation of HIV care, and PLHIV CD4^+^ T-cell counts at the start of HIV care and at ART initiation using data from the E-HIV database. For timeliness of PLHIV accessing HIV care, we looked at individuals newly diagnosed with HIV in the study year and calculated the time from HIV confirmation to linkage to HIV medical care for each patient. For this analysis, linkage to care was defined as the first visit to an infectious disease doctor (qualified to follow and treat people infected with HIV in Estonia) when a CD4^+^ T-cell count and/or HIV RNA level was measured. We considered linkage to HIV medical care to be timely when this first visit took place within 90 days of HIV confirmation [17]. For this calculation, to allow all patients newly diagnosed with HIV at least 90 days to access HIV medical care, only those newly diagnosed by 1 June 2013 were included. We also looked at E-HIV data on patients’ CD4^+^ T-cell counts obtained during the first HIV medical visit (as defined above) and at ART intiation.

Information bias in data sources was mitigated by detailed data review at face-to-face meetings of the research team and consultations with HIV medical care providers and community partners. We compiled cross-comparisons of the data, discussed any discrepancies and, if necessary, obtained additional data and consultations until consensus was reached.

## Results

### People living with HIV 

According to the most recent UNAIDS estimation there were 8,628 (range: 6,941–10,783) PLHIV in Estonia in 2013 [13].

### People diagnosed with HIV

A total of 8,605 new HIV cases were registered in Estonia from 1988 to 31 August 2013 according to the EHB: 6,909 in the period 1988 to 2008, and 1,696 in the period 2009 to 2013 [14]. Accounting for the potential 6–34% multiple registrations of new cases until 2009 [3], we estimated that the actual number of people diagnosed with HIV in Estonia over this time may have ranged from 6,242 to 8,164 (mean: 7,203).

According to the Estonian Causes of Death Registry (ECDR), 455 AIDS-related deaths were recorded up to 31 August 2013 (personal communication: G. Denissov, ECDR, 4 December 2103). According to the national drug information centre, there were 1,118 deaths by drug overdose in Estonia between 1999 and 2012 [20], and in 2013 (until 31 August 2013), an additional 81 such deaths (personal communication: G. Denissov, ECDR, 12 February 2015). Of these deaths, 88–94% could be attributed to injection drugs [20]. Thus, we estimated that between 1999 and 31 August 2013, between 1,055 ((1,118 + 81) × 0.88) and 1,127 ((1,118 + 81) × 0.94) deaths related to injection drug overdose may have occurred. However, we also had to take into account that not all those who died from the overdose would have been HIV-positive. According to data from local studies among PWID from 2005 to 2013, the proportion of PWID having ever tested HIV-positive ranged from 27 to 63% (data not shown). Considering that, we estimated that between 285 (1,055 × 0.27) and 710 (1,127 × 0.63) PLHIV may have died from injection drug overdose in Estonia in this period.

Taking into account multiple registration of newly diagnosed HIV cases, the AIDS-related deaths and the deaths due to injection drug overdose among PLHIV, we calculated that between 5,077 (6,242 − 455 − 710) and 7,424 (8,164 − 455 − 285) individuals diagnosed with HIV (mean: 6,251) were living in Estonia on 31 August 2013. Hence, altogether 72% of the 8,628 PLHIV estimated to live in Estonia in 2013 can be expected to have been diagnosed with HIV.

### PLHIV linked to HIV care

Since the inception of the EHIF electronic database of medical claims in 2000, altogether 4,375 HIV-positive patients (alive by the end of our study) had received at least one HIV-related medical service (with the HIV-specific ICD-10 code on the medical claim) by (or referred from) an infectious disease doctor, department or clinic. Thus, according to EHIF data, 51% of the 8,628 HIV-positive people estimated to be in Estonia in 2013 could be considered to have ever accessed HIV medical care by the end of our study.

### PLHIV retained in HIV care

In 2013, altogether 1,855 PLHIV (21% of the total 8,628 HIV-positive people estimated to live in Estonia) were considered ‘retained in care’ according to EHIF data.

### PLHIV on antiretroviral therapy 

In 2013, 1,250 PLHIV could have been considered ’retained in care’ according to E-HIV. Of those, 1,022 (82%) also received ART. Applying this proportion, we estimated that 1,521 (1,855 × 0.82) of the PLHIV ‘retained in care’ according to EHIF were also ’on ART’. This translates into 18% of the total 8,628 HIV-positive people estimated to live in Estonia in 2013.

### PLHIV on ART with suppressed viral load

E-HIV included at least one viral load test result during the study period for 1,021 of the 1,022 PLHIV continuously in care and on ART. Of these, 712 (70%) had achieved viral suppression (HIV RNA < 200 copies/mL) at their most recent test. We thus estimated that 1,065 (1,521 × 0.70) of PLHIV continuously in care and on ART had achieved viral suppression. This translates into 12% of the total 8,628 HIV-positive people estimated to live in Estonia in 2013.

All estimates for the steps of the HIV care cascade in Estonia in 2013 are summarised in Table 2.

**Table 2:** The HIV care cascade estimates in Estonia, 2013

People	Number of people	Proportion among PLHIV
Living with HIV	8,628 (6,941–10,783)	100%
Diagnosed with HIV (alive in 2013)	6,251 (5,077–7,424)	72% (47–100)
Linked to HIV care (alive in 2013)	4,375	51% (41–63)
Retained in HIV care	1,855	21% (17–27)
On ART	1,521	18% (14–22)
Virally suppressed	1,065	12% (10–15)

ART: antiretroviral therapy; HIV: human immunodeficiency virus; PLHIV: people living with HIV.

When looking at the timing of PLHIV linkage to HIV care, we found that according to E-HIV, 111 individuals were newly diagnosed with HIV during the study period (1 September 2012–31 August 2013). Excluding patients with missing data and allowing all patients 90 days to reach HIV care, we found that 86% of those newly diagnosed (74 patients of 86) had accessed HIV care within 90 days of testing HIV-positive.

Regardless of how long (up to 90 days or more) it had taken people diagnosed with HIV to access HIV medical care, more than half (62%) of the 90 individuals registered during the study period had a CD4^+^ T-cell count ≤ 350 cells/mm^3^ at registration as newly diagnosed with HIV. Considering data from E-HIV, we also found that majority of PLHIV starting ART (31 of 37 individuals) had had a CD4^+^ T-cell count ≤ 350 cells/mm^3^ at treatment initiation.

## Discussion

We found that in Estonia, as in other countries where engagement in HIV care has been evaluated [8-12,21], PLHIV are lost at each stage of the HIV care cascade; in 2013, only 12% of the total 8,628 HIV-positive people estimated to live in Estonia had achieved viral suppression. Engagement in different steps of HIV care in Estonia in 2013 resembles that recently described in Georgia [10], a country with similar political and economic history and HIV epidemic (driven by injection drug use until 2011) [22]. However, without unified standards for defining the stages of the cascade [12,23], PLHIV’s engagement in different stages of HIV care can only be compared between countries after carefully evaluating that similar definitions and methods of analysis and data sources have been used. 

Our results are less positive than a recent analysis based on expert opinion which suggested that 60% of PLHIV in Estonia in 2013 had seen an infectious disease specialist by 31 December 2013 (personal communication: M. Maimets, Tartu University Hospital/Estonian Society for Infectious Diseases, 26 July 2015) [13]. The difference from the 51% we calculated could derive from the expert analysis going back to the earliest (pre-epidemic) years of HIV in Estonia (1988–99) and including people who, although diagnosed and linked to care during that period, were not retained in care after 2000. In Estonia, antiretroviral drugs are distributed through a centralised system governed by the EMSA. According to the ministry, at the end of our study period (31 August 2013) 2,647 PLHIV were receiving ART (personal communication: E. Bauer, EMSA, 2 December 2013), representing 31% of all PLHIV estimated to live in Estonia in 2013 [13]. The difference from our estimate of 18% PLHIV on ART can be explained by different definitions used, as the EMSA figure represents cross-sectional prevalence, without retention in HIV care (as defined in our study) as a prerequisite. One might debate whether each step in the cascade should derive from the previous one(s), e.g. whether setting retention in care as a prerequisite for the following ‘on ART’ and ‘virally suppressed’ steps actually helps define PLHIV receiving proper care for HIV. In case ART data are easily available, as is the case in Estonia, one might be tempted to skip the ‘retained in care’ step, especially considering the recent developments in HIV medical care in the world. In 2014, UNAIDS set new targets to confront the HIV epidemic, focusing on four of the six steps in the classic cascade [24]. By the end of 2015, all major international HIV treatment guidelines, following the ‘test and treat’ approach, had introduced a recommendation to prescribe ART to all PLHIV upon diagnosis [25-28]. This should rapidly scale up ART distribution, and thus retention as an independent step in the HIV care cascade is likely to lose value. This also applies to Estonia, where the current guidelines from the European AIDS Clinical Society are followed [26] and persons living with HIV are treated irrespective of their CD4^+^ T-cell count. However, considering that retention would help evaluate the quality of HIV healthcare (other than ART) provided to PLHIV, monitoring retention would inform the national response to HIV. Although the Estonian National HIV/AIDS Prevention Strategy, which provided a framework for activities against HIV at the time of the study in 2013, ended in 2015 [4], the activities have been incorporated into the National Health Plan 2009–2020 and have continued [29,30]. 

According to our findings, the main gaps in PLHIV engagement in HIV care in Estonia in 2013 were that (i) about one quarter of the 8,628 persons estimated to live with HIV had not been diagnosed with HIV, (ii) another quarter, although aware of their HIV-positive serostatus, had not accessed HIV medical care and (iii) more than half of PLHIV, having accessed HIV medical care from an infectious disease specialist after diagnosis, were not retained in care. These findings highlight the need for continuous and enhanced effort to identify people with HIV for linkage and retention in care. Based on the absolute number of PLHIV concerned, the biggest issue were people not retained in care they had once been linked to. However, had we applied a more permissive definition for retention (e.g. utilisation of HIV healthcare services once a year in consecutive years), the second most important issue of PLHIV not tested for or not diagnosed with HIV would have become the biggest.

We found that the majority of PLHIV, diagnosed during the study period (1 September 2012–31 August 2013) had accessed specialised HIV care within three months of learning their HIV-positive status. However, this timely linkage occurred too late in the course of the disease, given the low CD4^+^ T-cell counts of PLHIV at initiation of specialist care (62% with ≤ 350 cells/mm^3^). Late linkage to HIV care seems to be related to delayed testing. In 2013, 52% of PLHIV in Estonia had a CD4^+^ T-cell count below 350 cells/mm^3^ when newly diagnosed, compared with the EU/EEA average of 47% [1], indicating the need to prioritise and intensify HIV testing policies and procedures in Estonia [31]. According to national recommendations, HIV testing is mandatory for blood and organ donors (and in some cases for people in the armed forces) and recommended for pregnant women, prisoners, people with hepatitis, tuberculosis, sexually transmitted dieases and a history of injection drug use or engagement in risky sexual behaviours [3], but screening only these target groups could be insufficient. Therefore local and national guidelines recommend routine HIV screening for all patients aged 16–49 years in healthcare facilities (except in emergency care, where clinical indications apply) in two counties most affected by HIV in Estonia [32]. However, healthcare providers experience challenges in implementing this policy due to lack of training, support and financial resources [31]. All testing programmes and centres should introduce guidelines and have pathways to ensure that people testing positive for HIV get linked to appropriate care [31]. In addition to implementing routine testing, emphasis on groups most at risk of acquiring HIV would facilitate earlier diagnosis [31]. A recent study among PWID, the key risk population in Estonia, revealed that about half of them had not been tested for HIV in the past year [33]. Introducing HIV testing in settings frequently attended by PWID (i.e. needle and syringe exhange sites) could scale up HIV testing among PWID in Estonia.

Our study also showed that more than half of PLHIV who accessed HIV medical care at least once after being diagnosed with HIV were not retained in care in 2013. This gap indicates the need to monitor the quality of HIV healthcare in Estonia, and to retain all steps (e.g. retention in care) in the cascade. Acknowledging the high proportion of PLHIV with current or past drug addiction in Estonia, the recent World Health Organization (WHO) evaluation report on HIV/AIDS treatment and care in the country highlighted the need to expand provision of integrated HIV and related services (e.g. antiretroviral and opioid substitution therapy) as an opportunity to improve HIV care [31].

Our study has several limitations related to measurements at each stage of the cascade of care. The true number of those infected with HIV in Estonia is not known, and we therefore used an estimate derived from UNAIDS. Estimates of the HIV care cascade are very sensitive to the HIV prevalence estimate. After our study was conducted, the WHO evaluation in 2014, based on a crude estimate for the number of people living with undiagnosed HIV in Estonia, suggested that the number of PLHIV might have been around 13,500 [31] instead of the UNAIDS estimate of 8,628 used in our study [13]. In 2017, the Estonian National Institute for Health Development (NIHD) will initiate a project to estimate the number of PLHIV in Estonia (personal communication: K. Rüütel, NIHD, 16 October 2016), using the HIV modelling tool from the European Centre for Disease Prevention and Control (ECDC) [34]. Future research into the HIV care cascade should weigh all the available estimates.

Our analysis is based on unlinked (between databases) and aggregated data and therefore might lack precision. The different sources of data about PLHIV and services used were established at different times, for different purposes, by different institutions. Data availability was the main obstacle to mapping HIV care, as also recognised by other researchers [35]. Missing data on HIV-positive people having died for causes other than AIDS or drug overdose are likely to lead to an overestimate for PLHIV in Estonia in 2013. On the other hand, although we applied carefully constructed case-finding algorithms to identify HIV-positive individuals from the health administrative databases, some cases may have been misclassified, causing an underestimation of HIV care coverage. However, we believe that nationwide coverage of the EHIF, and the EHB strengthen our analysis. In particular, EHIF data (used to derive population-based estimates on medical care linkage, retention and ART coverage) is considered to be representative of medical services provided in Estonia, as EHIF reimburses healthcare providers on a fee-for-service basis. However, none of the databases includes all the information needed to characterise PLHIV at all the stages of HIV care in Estonia, and therefore several assumptions had to be made. Further, for some of the HIV care coverage estimates (the proportion of PLHIV on ART, virally suppressed), we extrapolated data from E-HIV to EHIF data to obtain population-based estimates. Although we are not aware of studies assessing the coverage of E-HIV and factors associated with inclusion in E-HIV (e.g. clinical factors, healthcare utilisation), we might speculate that E-HIV-based proportions are overestimates of those on ART who are virologically suppressed.

Summarising the indicators for the different stages in HIV medical care in Estonia in the format of the well-known HIV treatment cascade [8,9,33] allows easier comparisons between countries of PLHIV engagement in HIV care. However, keeping in mind all the assumptions we had to make during the analysis, such a summary probably gives a simplified picture of the situation in Estonia. It should also be remembered that the point estimates for the number of PLHIV at each step, starting from the UNAIDS estimate for the number of PLHIV in Estonia, include a range, and the ranges of several consecutive estimates overlap.

Our study, identifying the main gaps in connecting PLHIV to sustained and quality care should support policymakers and service providers in Estonia in enhancing services and systems that best support PLHIV as they move through the continuum of HIV medical care.
